# Empirical Variational Mode Decomposition Based on Binary Tree Algorithm

**DOI:** 10.3390/s22134961

**Published:** 2022-06-30

**Authors:** Huipeng Li, Bo Xu, Fengxing Zhou, Baokang Yan, Fengqi Zhou

**Affiliations:** 1 Engineering Research Center for Metallurgical Automation and Measurement Technology, Ministry of Education, Wuhan University of Science and Technology, Wuhan 430081, China; yehuip@hgnu.edu.cn (H.L.); zhoufengxing@wust.edu.cn (F.Z.); yanbaokang@wust.edu.cn (B.Y.); zhoufengqi@wust.edu.cn (F.Z.); 2 School of Physics and Electronic Information, Huanggang Normal University, Huanggang 438000, China

**Keywords:** non-stationary signal, empirical variational mode decomposition, binary tree, least square mutual information, information entropy

## Abstract

Aiming at non-stationary signals with complex components, the performance of a variational mode decomposition (VMD) algorithm is seriously affected by the key parameters such as the number of modes K, the quadratic penalty parameter α and the update step τ. In order to solve this problem, an adaptive empirical variational mode decomposition (EVMD) method based on a binary tree model is proposed in this paper, which can not only effectively solve the problem of VMD parameter selection, but also effectively reduce the computational complexity of searching the optimal VMD parameters using intelligent optimization algorithm. Firstly, the signal noise ratio (SNR) and refined composite multi-scale dispersion entropy (RCMDE) of the decomposed signal are calculated. The RCMDE is used as the setting basis of the α, and the SNR is used as the parameter value of the τ. Then, the signal is decomposed into two components based on the binary tree mode. Before decomposing, the α and τ need to be reset according to the SNR and MDE of the new signal. Finally, the cycle iteration termination condition composed of the least squares mutual information and reconstruction error of the components determines whether to continue the decomposition. The components with large least squares mutual information (LSMI) are combined, and the LSMI threshold is set as 0.8. The simulation and experimental results indicate that the proposed empirical VMD algorithm can decompose the non-stationary signals adaptively, with lower complexity, which is O(*n*^2^), good decomposition effect and strong robustness.

## 1. Introduction

Many physical semaphores in real life are composed of multi-components, which have nonlinear and nonstationary characteristics. It is an essential way to explore the system characteristic by analyzing the inherent information contained in the components. Due to the interference of the external environment, these characteristic components are difficult to effectively identify. Consequently, the effective extraction of these signal modes becomes very important for the research of corresponding systems. The methods of signal or data processing have attracted more and more attention in various fields.

In recent years, many non-stationary signal processing methods have been proposed by scholars. Short time Fourier transform (STFT) is a traditional time-frequency transform (TF) method [1]. It provides a graphic display in the TF domain and has been successfully applied to the evaluation of mechanical fault characteristics [2]. STFT is restricted by the time-frequency resolution of window function, and the time-frequency resolution is fixed. Wavelet transform (WT) [3] has high-precision time-frequency resolution, and the selection of the WT basis function has a huge impact on performance and has strong experience [4]. Wigner Ville Distribution (WVD) [5] is a method to calculate the time–frequency distribution. It can reflect the instantaneous time–frequency relationship of a signal [6]. However, it is seriously affected by cross terms. S-transform (ST) [7] combines the advantages of STFT and WT, but its spectrum is rough and restricted by the Heisenberg’s uncertainty principle [8]. In theory, high-order statistics (HOS) [9] can completely suppress Gaussian noise, but it has poor effect on non-Gaussian noise and interferes with the high-order spectrum of the signal. Empirical mode decomposition (EMD) [10] is a method to decompose signals into a set of intrinsic mode functions (IMF). It is an excellent adaptive signal processing method and has been widely used in engineering [11,12]. However, there are some problems such as fitting overshoot, endpoint effect and modal aliasing, which seriously restrict its practical application [13]. Local mean decomposition (LMD) [14] adaptively decomposes the non-stationary multi-component signal into the sum of several product functions (PF) with physical meaning of instantaneous frequency [15]. It has the disadvantages of signal mutation and large amount of calculation caused by demodulation [16]. Inherent time scale decomposition (ITD) [17] suffers from the problem that the waveform of PF component fluctuates locally, resulting in signal distortion [18]. Although many scholars put forward a large number of improvement methods to the above method, they also gained good results and successfully applied them to mechanical fault diagnosis. Nevertheless, confined by the theoretical framework, these problems can be suppressed to a certain extent, but cannot be fundamentally excluded.

VMD [19] is a new adaptive signal processing method, which decomposes the signal into a set of modal functions with limited bandwidth by iteratively solving the variational problem. It realizes the frequency separation of each signal component and overcomes the problems of endpoint effect, modal mixing and waveform fluctuation existing in EMD, LMD and ITD. This strategy is very suitable for analyzing nonlinear and non-stationary vibration signals, and has been widely used in the engineering field [20,21]. However, the performance of VMD is affected by the inherent decomposition parameters i.e., the total number of modes K, the quadratic penalty parameter α, update step τ. Moreover, these parameters must be preset and have strong artificial experience. VMD converts signal decomposition into a constrained variational problem and adaptively decomposes the signal into the sum of several Intrinsic Mode Function (IMF) components, which is essentially different from previous signal processing methods [22]. However, the key parameters must be artificially set in advance, and these parameters have a great influence on the decomposition results. The most important thing is that there is no standard to measure the result of decomposition. Therefore, in practical application, the process of artificially setting parameter values in advance indicates that it is not a fully adaptive model [23,24]. In recent years, many scholars have conducted relevant research on VMD parameters setting. Xiao et al. [25] proposed an optimal value search method for the decomposition parameters (α and K) of VMD. However, the search method relies on personal experience and intuitive search mechanism, and lacks the basis of mathematical theoretical framework. Kaur et al. [26] used discrete wavelet transform (DWT) and wavelet packet transform (WPT) to set the VMD decomposition mode number. However, under the strong noise interference, it is a great challenge to obtain the modal number. Long et al. [27] applied the center frequency observation method to set the number of modes, and modified the parameter value τ according to the residual index to verify the influence of the parameter on the signal decomposition. However, the method of screening single parameter value based on single index does not take into account the interaction between parameters. Considering the complexity and variability of actual vibration signals, the application of this observation method in engineering may be limited. Recently, some studies have proposed optimization algorithms to adjust the parameters. In Ref. [28], particle swarm optimization (PSO) was adopted to select the optimal combination value of decomposition parameters K and τ of VMD. This method alleviates the experience of setting parameters manually to a certain extent. Wang et al. [29] obtained the optimal selected parameters, namely the mode number and penalty parameter of VMD, by using a PSO optimization algorithm through the appropriate fitness function. Xu et al. [30] applied the variable dimension composite chaotic algorithm to adaptively select parameters of VMD and obtained excellent performance. However, the complexity of the algorithm and the feasibility of practical application need to be verified. A new method termed variational mode extraction (VME) extracts the natural mode function by knowing its approximate center frequency [31], which can adaptively extract the modal components in the signal. However, the residual signal in its decomposition has no strict mathematical definition and physical significance. The improved VME method is named Successive VMD (SVMD) in [32,33]. The SVMD achieves good adaptive effect, but the residual signal still lacks strict physical definition. The mentioned methods have improved VMD performance and achieved satisfactory effect to some extent. However, the synergistic influence of VMD key parameters has not been fully considered.

In view of the above issues, this paper proposes an empirical VMD method based on the binary tree model. Its main innovations and contributions can be summarized as follows:

(1) The number of modes K in a traditional VMD needs to be manually set. In this paper, K is set as a fixed value, that is, K = 2, which can effectively avoid empirically setting the value of K. Then the decomposition is iteratively executed according to the binary tree model until the single component of the decomposition is duplicated. Lastly, according to the mutual information between each component, the components with larger mutual information value are added to obtain the new IMF component.

(2) The α of traditional VMD still needs to be manually set. This paper presents a calculation equation of α, namely α = *round*(RCMDE × (*f_s_*/2) ∗ log(*K*)). Where *f_s_* is the sampling frequency of the signal, round(⋅) is the rounding function, RCMDE is the refined composite multi-scale dispersion entropy. RCMDE can measure the complexity of signal well and adjust the value of α dynamically.

(3) The τ critically affects the convergence of VMD. Hence, it is required to select an appropriate τ value according to the noise level of the signal to guarantee the optimal convergence of the algorithm. In this paper, the signal to noise ratio (SNR) is proposed to dynamically set the τ value, which can effectively guarantee the convergence and convergence speed of the algorithm.

(4) The algorithm proposed in this paper fully considers the common influence of several key parameters of VMD and is adaptive. Compared with the improved method based on the intelligent optimization algorithm, its computational complexity is lower.

Lastly, the effectiveness and superiority of the proposed method are verified by analyzing the simulation signal and the measured vibration signal. 

The rest of this paper is organized as follows. The Section 2 introduces the relevant basic theoretical knowledge in detail. The Section 3 introduces the empirical VMD algorithm structure based on binary tree model in detail. In the Section 4, the effectiveness of the proposed algorithm is experimentally verified. Finally, the conclusion is drawn in the Section 5.

## 2. Related Works

### 2.1. Brief Introduction of VMD

VMD is an adaptive signal decomposition method based on Wiener filter, Hilbert transform and heterodyne demodulation. Its purpose is to decompose a real-valued input signal x(t) into a set of sub-modes $\left\{u_{k}\right\}$ with a particular sparsity. Each of the limited bandwidth sub-modes is tightly centered around a central frequency $\omega_{k}$. In the process of VMD, each submode $u_{k}$ is transformed by Hilbert transform to obtain the corresponding unilateral spectrum. The analytical signal of each component is mixed with a pre-estimated center frequency $e^{-j\omega_{k}t}$ and the spectrum of each mode is modulated to the corresponding base-band. In order to obtain the bandwidth of the sub-modes, the constraint variational problem is introduced to calculate the square $L^{2}$-norm of the gradient of the demodulated signals and estimate the bandwidth of each mode. The optimized variational model constructed is shown in Equation (1).

(1) $$\left\{ \begin{array}{l} \underset{\left\{u_{k}\},\{\omega_{k}\right\}}{\min}\left\{\sum_{k=1}^{K}{\left\|\partial_{t}\left[\left(\delta (t)+\frac{j}{\pi t}\right)*u_{k}(t)\right]e^{-j\omega_{k}t}\right\|}_{2}^{2}\right\}\\ s.t.\begin{array}{cc} & \sum_{k=1}^{K}u_{k}(t)=x(t) \end{array} \end{array}\right.$$

where, K is the number of mode components, $\left\{u_{k}\}=\{u_{1},u_{2},\cdots ,u_{K}\right\}$ and $\left\{\omega_{k}\}=\{\omega_{1},\omega_{2},\cdots ,\omega_{K}\right\}$ are components and corresponding central frequency respectively. $\sum_{k}:=\sum_{k=1}^{K}$ is equivalent to the sum of all band components.

#### 2.1.1. Solution of Variational Problem

By introducing the quadratic penalty factor α and the Lagrange multiplication operator λ(t), the constrained variational problem is transformed into a non-constrained variational problem. The quadratic penalty factor can guarantee the signal reconstruction accuracy under the condition of Gaussian noise, and the Lagrange multiplier emphasizes the strictness of constraints.

(2) $$\begin{array}{l} L\left(\{u_{k}\},\{\omega_{k}\},\lambda \right):=\alpha {\sum_{k}\left\|\partial t\left[\left(\delta (t)+\frac{j}{\pi t}\right)*u_{k}(t)\right]e^{-j\omega_{k}t}\right\|}_{2}^{2}\\ {\begin{array}{cc} & +\left\|f(t)-\sum_{k}u_{k}(t)\right\| \end{array}}_{2}^{2}+\langle \lambda (t),f(t)-\sum_{k}u_{k}(t)\rangle \end{array}$$

Then, the $\left\{u_{k}^{n+1}\right\}$, $\left\{\omega_{k}^{n+1}\right\}$ and $\lambda^{n+1}$ are updated alternately by using the alternate direction method of multipliers (ADMM). By seeking the ‘saddle point’ of the extended Lagrange expression, that is, meeting the stop condition of iteration, mutually independent frequency band components $\left\{u_{k}\right\}$ are finally concluded. The decomposition process of the variational model is summarized in Algorithm 1.
**Algorithm 1:** Complete optimization of VMD**Initialize:**$\left\{{\hat{u}}_{k}^{1}\right\}$,$\left\{\omega_{k}^{1}\right\}$,${\hat{\lambda }}^{1}$,n←0 **repeat:**  n←n+1   **for**k=1:K**do**  Update ${\hat{u}}_{k}$ for all ω≥0**:**  ${\hat{u}}_{k}^{n+1}(\omega )\leftarrow \frac{\hat{f}(\omega )-\sum_{i<k}{\hat{u}}_{i}^{n+1}(\omega )-\sum_{i>k}{\hat{u}}_{i}^{n}(\omega )+\frac{{\hat{\lambda }}^{n}(\omega )}{2}}{1+2\alpha {\left(\omega -\omega_{k}^{n}\right)}^{2}}$     (3)  Update $\omega_{k}$**:**  $\omega_{k}^{n+1}\leftarrow \frac{\int_{0}^{\infty }\omega {\left|{\hat{u}}_{k}^{n+1}(\omega )\right|}^{2}d\omega }{\int_{0}^{\infty }{\left|{\hat{u}}_{k}^{n+1}(\omega )\right|}^{2}d\omega }$                 (4)   **end for**  Dual ascent for all ω≥0**:**  ${\hat{\lambda }}^{n+1}(\omega )\leftarrow {\hat{\lambda }}^{n}(\omega )+\tau \left(\hat{f}(\omega )-\sum_{k}{\hat{u}}_{k}^{n+1}(\omega )\right)$    (5)   **until** convergence: ${\sum_{k}\left\|{\hat{u}}_{k}^{n+1}-{\hat{u}}_{k}^{n}\right\|}_{2}^{2}/{\left\|{\hat{u}}_{k}^{n}\right\|}_{2}^{2}<$*ϵ*   (6)

More detailed description of the VMD algorithm can refer to Ref. [19].

#### 2.1.2. Parameter Influence Analysis

The process of solving the variational model shows that the performance of VMD is closely related to the intrinsic parameters [34], such as the total number of modes K, the quadratic penalty α, the update step τ, and the convergence fault tolerance threshold ε. The influence of each parameter on the decomposition performance of VMD is analyzed as follows.

The performance of VMD is very sensitive to the value of K. If K is set too small, the signal will be under-segmented and some components will be included in other modes. On the contrary, the high value of K will cause mode duplication and other problems.

Parameter α is related to the performance of suppressing noise interference. A large value of α may result in a narrow bandwidth of modal components, and some information may be lost in the original signal. An α value that is too small will lead to too large of a bandwidth of modal components, some components will be included in other modes or extra noise will be captured.

When the noise level of the signal is low, the Lagrangian multiplier can ensure the optimal convergence by choosing the appropriate value of *τ* (*τ* > 0). Accordingly, when the noise level of the signal is high, the Lagrangian multiplier will seriously hinder the convergence of the algorithm if *τ* > 0. Setting *τ* = 0 can effectively turn off the Lagrangian multiplier to ensure the effective convergence of the algorithm.

The value of convergence tolerance ε will affect the reconstruction accuracy of VMD decomposition. The reconstruction error (RSE) can be controlled by reducing the convergence of stop criterion to a certain extent.

From the above analysis, the above four key parameters seriously affect the performance of VMD. In addition, the interaction between parameters will affect the algorithm’s performance. Therefore, choosing the right combination of VMD parameters is the key factor to determine its performance.

### 2.2. Least Squares Mutual Information

Mutual information [35,36,37] is a nonparametric and nonlinear measure index in information theory that can quantitatively express the correlation between two random variables and is more accurate than the correlation coefficient method [38]. According to the principle of irrelevance and orthogonality equivalence between zero mean random signals, mutual information can measure the coupling degree between Intrinsic Mode Function (IMF) components and residual information obtained in VMD decomposition. In other words, mutual information can measure whether modal mixing occurs and the determine its degree. Mutual information is defined as follows:

(7) $$\mathrm{MI}=\frac{1}{2}\int \sum_{y=1}^{c}p(x,y)\log\frac{p(x,y)}{p(x)p(y)}dx$$

where p(x,y) is the joint probability density function of signals x and y, p(x) and p(y) are marginal probabilities distribution, respectively. In Equation (7), the response of the logarithm function in mutual information to outliers fluctuates greatly, which affects the accuracy of estimation. Therefore, in order to overcome this problem, in this paper, the square loss mutual information is induced to replace the logarithmic function, so as to reduce the interference of outliers and obtain more accurate mutual information estimator. The definition of substitution is as follows:

(8) $$\mathrm{MI}=\frac{1}{2}{\int \sum_{y=1}^{n}p(x)p(y)\left(\frac{p(x,y)}{p(x)p(y)}-1\right)}^{2}dx$$

Avoiding the calculation of joint probability p(x,y), marginal probability p(x), and marginal probability p(y), the least square estimation method is introduced to calculate the mutual information of the square loss, and the combined density ratio function is learned directly. The density ratio function is defined as follows:

(9) $$\omega (x,y)=\frac{p(x,y)}{p(x)p(y)}$$

By taking the Gaussian radial basis kernel (RBF) model related to the parameters, the density ratio function can be approximated as:

(10) $$\omega_{\alpha }(x,y)=\sum_{j=1}^{n}\alpha_{j}\exp\left(-\frac{{\left\|x_{j}-y_{j}\right\|}_{2}^{2}}{2h^{2}}\right)$$

where $\alpha \;=\;(\alpha_{1},\cdots ,\alpha_{n}{)}^{T}$ is the parameter vector. Gaussian radial basis kernel is selected as the basis function ψ. Then, the least square learning is performed for the parameter α corresponding to the minimum of the following J(α).

(11) $$\begin{array}{l} J(\alpha )=\frac{1}{2}{\int \sum_{y=1}^{n}\left(\omega_{\alpha }(x,y)-\omega (x,y)\right)}^{2}p(x)p(y)dx\\ \;\;=\frac{1}{2}\int \sum_{y=1}^{n}\alpha^{T}\psi (x,y)\psi {\left(x,y\right)}^{T}\alpha p(x)p(y)dx\\ \;\;-\int \sum_{y=1}^{n}\alpha^{T}\psi (x,y)p(x,y)dx+C \end{array}$$

where the third term $C=\frac{1}{2}\int \sum_{y=1}^{n}\omega (x,y)p(x,y)dx$ is a constant independent of parameters, which can be ignored in the calculation. By using the sample average approximation of the expected values contained in the first and second terms of Equation (11) and introducing the $L^{2}$ regularization term, the learning rule can be derived as:

(12) $$\underset{\alpha }{\min}\left[\frac{1}{2}\alpha^{T}\hat{G}\alpha -\alpha^{T}\hat{h}+\frac{\lambda }{2}{\left\|\alpha \right\|}^{2}\right]$$

(13) $$\left\{ \begin{array}{l} \hat{G}=\frac{1}{n^{2}}\sum_{i,i^{\prime }}^{n}\psi (x_{i},y_{i^{\prime }})\psi {\left(x_{i},y_{i^{\prime }}\right)}^{T}\\ \hat{h}=\frac{1}{n}\sum_{i=1}^{n}\psi (x_{i},y_{i}) \end{array}\right.$$

where $\hat{G}$ is a matrix with n×n order and $\hat{h}$ is a n dimensional vector. The learning rule is a convex quadratic form related to α, and its optimization problem is as follows:

(14) $$\hat{\alpha }:=\underset{\alpha }{\arg\min}\left[\frac{1}{2}\alpha^{T}\hat{G}\alpha -\alpha^{T}\hat{h}+\frac{\lambda }{2}{\left\|\alpha \right\|}^{2}\right]$$

The analytical solution can be obtained by taking the derivatives of Equation (14) and solving it equal to zero, namely,

(15) $$\hat{\alpha }=(\hat{G}+\lambda I{)}^{-1}\hat{h}$$

where λ is the regularization parameter and I is the identity matrix. The density ratio estimator obtained by the above method is substituted into Equation (16), which is equivalent to the square loss mutual information.

(16) $$\mathrm{MI}=\frac{1}{2}\int \sum_{y=1}^{n}\omega (x,y)p(x,y)dx-\frac{1}{2}$$

Then, the least squares mutual information (LSMI) estimation is obtained as follows:

(17) $$\mathrm{LSMI}=\frac{1}{2}{\hat{h}}^{T}{\left(\hat{G}+\lambda I\right)}^{-1}\hat{h}-\frac{1}{2}$$

where the regularization parameter λ and the parameters contained in the basis function ψ can be determined by the optimization algorithm related to rule J.

### 2.3. Refined Composite Multi-Scale Dispersion (RCMDE)

Information entropy [39] is an indicator that measures the uncertainty of information quantity, which represents the average uncertainty of a signal. In information theory, information entropy is used to measure the amount of information. The larger the information entropy, the greater the uncertainty and the complexity of the signal present. At present, information entropy is widely used in the field of mechanical fault diagnosis and medical diagnosis, and has achieved fruitful research results. The commonly used information entropy primarily includes approximate entropy, sample entropy, permutation entropy and so on. Many achievements have been made in the application of these techniques in signal nonlinear feature extraction. Sample entropy is characterized by large computation and slow computation, and permutation entropy does not take into account the differences between vibration amplitudes. Rostaghi and Azami [40] proposed a new method to measure the complexity of time series, namely dispersion entropy (DE), which solves the shortage of sample entropy and permutation entropy to some extent. Furthermore, Azami [41] proposed the fine composite multi-scale dispersion entropy (RCMDE) method, which has the characteristics of good stability in multi-scale process.

#### 2.3.1. Dispersion Entropy

(1) The normal distribution function (NDF) is used to map the time series $x=\left\{x_{j}|j=1,2,\cdots ,N\right\}$ to $y=\left\{y_{j}|j=1,2,\cdots ,N\right\}$ y∈(0,1). *N* denotes the length of the sequence. The mapping function is as follows:

(18) $$y_{j}=\frac{1}{\sqrt{2\pi }\sigma }\int_{-\infty }^{x_{j}}e^{\frac{-{\left(t-\mu \right)}^{2}}{2\sigma^{2}}}dt$$

where μ and σ represent the expectation and standard deviation respectively.

(2) Map y to integers in the range [1, C] using a linear transformation.

(19) $$z_{j}^{c}=round(cy_{j}+0.5)$$

where round(⋅) is the rounding function, and $z_{j}^{c}$ is the *j*-th element of classification sequence $z^{c}$.

(3) Calculate the embedding vector as follows:

(20) $$z_{i}^{m,c}=\left[z_{i}^{c},z_{i+d}^{c},\cdots ,z_{i+(m-1)d}^{c}\right],i=1,2,\cdots ,N-(m-1)d$$

(21) $$z_{i}^{c}=v_{0},\;z_{i+d}^{c}=v_{1},,\;z_{i+(m-1)d}^{c}=v_{m-1}$$

where m is the embedded dimension. d is the time delay.

(4) The dispersion pattern corresponding to each $z_{i}^{m,c}$ is $r_{v_{0},v_{1}\cdots v_{m-1}}$.$z_{i}^{m,c}$. It contains m digits in total, and each digit has c values. Therefore, the total number of dispersion modes of $z_{i}^{m,c}$ is $c^{m}$.

(5) The probability of each dispersion mode t can be defined as:

(22) $$p\left(r_{v_{0}v_{1}\cdots v_{m-1}}\right)=\frac{N\left(r_{v_{0}v_{1}\cdots v_{m-1}}\right)}{N-(m-1)d}$$

where $N\left(r_{v_{0},v_{1}\cdots v_{m-1}}\right)$ is the number of dispersion modes corresponding to $z_{i}^{m,c}$.

According to the definition of information entropy, the dispersion entropy can be expressed as:

(23) $$DE\left(x,m,c,d\right)=-\sum_{r=1}^{c^{m}}p\left(r_{v_{0}v_{1}v\cdots v_{m-1}}\right)\ln\left(p\left(r_{v_{0}v_{1}v\cdots v_{m-1}}\right)\right)$$

where r represents the type of dispersion mode corresponding to $z_{i}^{m,c}$. From the calculation method of dispersion entropy, it can be seen that when the probabilities of all dispersion modes are equal, the dispersion entropy has the maximum value $\ln(c^{m})$. The greater the dispersion entropy is, the higher the unpredictable degree of time series denotes, and vice versa.

#### 2.3.2. Multi-Scale Dispersion Entropy

The multi-scale dispersion entropy (MDE) proposed on the basis of dispersion entropy can reflect the complexity of time series at different scales. The calculation method of multi-scale dispersion entropy is as follows:

Firstly, The original signal $u=\left\{u_{1},u_{2},\cdots ,u_{L}\right\}$ with length *L* is roughened to obtain *N* sequences with length τ, and the coarse-grained signal is obtained by calculating the average value of each sequence.

(24) $$x_{j}^{\tau }=\frac{1}{\tau }\sum_{b=(j-1)\tau +1}^{}u_{b},1\leq j\leq \left\lfloor \frac{L}{\tau }\right\rfloor$$

where ⌊L/τ⌋ represents the length of each coarse-grained time series.

Secondly, calculate the dispersion entropy $DE\left(x^{\tau },m,c,d\right)$ of coarse-grained signal under each scale factor τ.

Finally, the MDE index can be obtained, as shown in the following:

(25) $$MDE\left(x,m,c,d,\tau \right)=\frac{1}{\tau }\sum_{i=1}^{\tau }DE\left(x^{\tau },m,c,d\right)$$

#### 2.3.3. Refined Composite Multi-Scale Dispersion Entropy (RCMDE)

For time series with different scales, parameter τ actually corresponds to different starting points of coarsening process. RCMDE value is defined as the average value of dispersion entropy of coarsening sequence. The *k*-th coarsening sequence of signal $u=\left\{u_{1},u_{2},\cdots ,u_{L}\right\}$ is:

(26) $$x_{k}^{\tau }=\left\{x_{1}^{\tau },x_{2}^{\tau },\cdots ,x_{L}^{\tau }\right\}$$

(27) $$x_{k}^{\tau }=\frac{1}{\tau }\sum_{b=k+\tau (j-1)}^{k+\tau j-1}u_{b},1\leq j\leq \left\lfloor \frac{L}{\tau }\right\rfloor ,1\leq k\leq \tau$$

The RCMDE value under scale τ. is calculated as follows:

(28) $$E_{RCMD}\left(x,m,c,d,\tau \right)=-\sum_{r=1}^{c^{m}}\bar{p}\left(r_{v_{0}v_{1}v\cdots v_{m-1}}\right)\ln\left(\bar{p}\left(r_{v_{0}v_{1}v\cdots v_{m-1}}\right)\right)$$

where $E_{RCMD}\left(x,m,c,d,\tau \right)$ is the RCMDE value under scale τ. $\bar{p}\left(r_{v_{0}v_{1}v\cdots v_{m-1}}\right)$ is the average value of dispersion mode probability corresponding to coarsening sequence, as follows:

(29) $$\bar{p}\left(r_{v_{0}v_{1}v\cdots v_{m-1}}\right)=\frac{1}{\tau }\sum_{k=1}^{\tau }p_{k}^{\tau }$$

where $p_{k}^{\tau }$ is the probability of the dispersion pattern corresponding to the *k*-th coarsening sequence under scale τ.

### 2.4. Efficient Signal Evaluation Index

Generally speaking, the observation signal consists of effective signal and noise components. To verify the noise robustness of the proposed method, a kind of metric, namely, the signal noise ratio (SNR), was applied and may be defined as follows:

(30) $$SNR=10\log\left\{\frac{\sum_{i=1}^{L}x^{2}(i)}{\sum_{i=1}^{L}({\left(x(i)-\hat{x}(i)\right)}^{2}}\right\}$$

where x(i) is the observed signal, $\hat{x}(i)$ is the mean value of x(i), and L denotes the length of x(i).

### 2.5. Analysis of Intrinsic Mode Function

At present, many signal decomposition methods take the orthogonality between modal components as the stop condition of decomposition, including many improved VMD algorithms. However, the orthogonality between IMF components could not guarantee the unity of IMF component characteristics. According to the decomposition principle of standard VMD, each IMF component has a single characteristic. A test was executed to demonstrate the orthogonality between IMF components and its own singleness.

**Figure 1:**
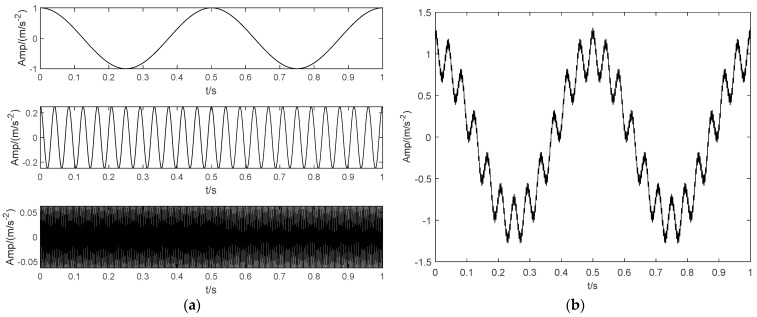

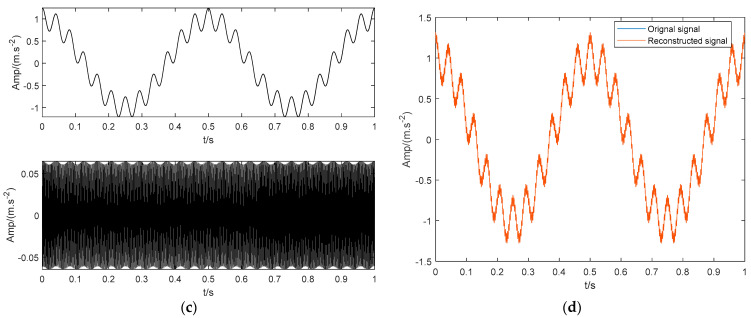
The singularity analysis of IMF components. (**a**) Sinusoidal harmonics. (**b**) Mixed signal. (**c**) Modes. (**d**) The reconstructed signal.

**Figure 2:**
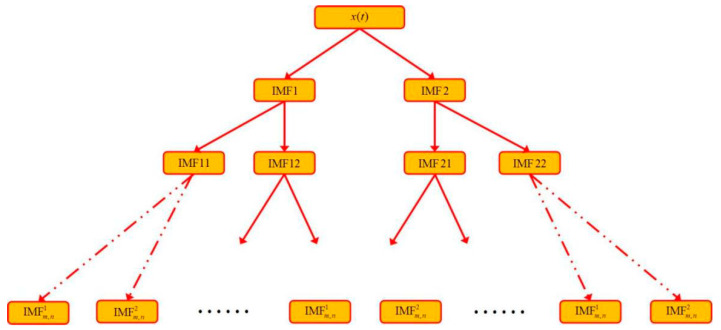
Diagram of binary tree decomposition.

As shown in Figure 1a, three harmonic signals are selected, and their corresponding frequencies are 2 Hz, 24 Hz and 288 Hz, respectively. The three harmonics are linearly superimposed to obtain the mixed signal, as shown in Figure 1b. Then, the original VMD is used to decompose the mixed signal. The preset parameters of VMD are K = 2, α = 2000. The decomposition result is shown in Figure 1c. The LSMI between the two IMF components (IMF1 and IMF2) in Figure 1c is 1.0 × 10^−6^, which is approximately orthogonal. However, it can be clearly observed that the IMF1 component contains two harmonic components (2 Hz and 24 Hz). It is not a single modal function. Finally, the IMF1 and IMF2 components are combined to reconstruct the representation signal, and the results are shown in Figure 1d. Compared with the original mixed signal, the reconstructed error is 1.0 × 10^−10^. Experimental results indicate that, although the orthogonal coefficient and reconstruction error index between IMF components are very small, it cannot fully ensure the unity of IMF components. On the one hand, it shows that the ideal built-in parameters of VMD have a great influence on the decomposition. On the other hand, VMD is needed to deal with the under decomposition and over decomposition of components adaptively. Therefore, this paper proposes a decomposition method based on the binary tree mechanism as shown in Figure 2, which can effectively ensure the unity of the IMF component.

## 3. Proposed Algorithm Framework

In this research work, an empirical VMD algorithm based on a binary tree model (BT-EVMD) is presented, which can effectively solve the problem of selecting the key parameters of VMD and make the decomposition process of VMD completely an adaptive process. The detailed procedure is as follows:

Step 1: Calculate the signal to noise ratio (SNR) and RCMDE of the original non-stationary signal x(t), and initialize key parameters of VMD as K = 2, α = RCMDE × round((*f_s_*/2) ∗ log(K)) (*f*_s_ is the sampling frequency and round(⋅) is the rounding function), τ = SNR and ε = 1 × 10^−7^. The signal is decomposed by VMD to obtain two IMF components, called IMF1 and IMF2.

Step 2: Initializes the kernel parameters of the Gaussian radial basis function of LSMI. Preset the threshold δ of LSMI estimator $LMSI_{E}$ and the threshold ρ of reconstruction error (RSE, the error between the sum of decomposed modes and input signal).

Step 3: Compute the LSMI of IMF1 and IMF2 (if $LMSI_{E}$ = 0, there is no similar information between IMF components, if $LMSI_{E}$ = 1, the information between IMF components is exactly the same, $0\leq LMSI_{E}\leq 1$). Determine whether $LMSI_{E}$ is greater than the threshold. If yes, end the decomposition. Else, calculate the reconstruction error ρ. If ρ > 1 × 10^−7^, terminate the process. Else, take the decomposed IMF components as two new signals, and repeat the Step 1 to continue the iteration.

Step 4: The LSMI between each IMF is computed. A new IMF component is obtained by adding the IMFs that meet $LMSI_{E}$ > δ.

After the decomposition of BT-EVMD, the multi-component and non-stationary signal can be adaptively decomposed into several sub-components.

The schematic diagram of decomposition is shown in Figure 2.

## 4. Experiment Validations

### 4.1. Simulation Analysis

In order to more clearly verify the effectiveness of the proposed method. This section applies the BT-EVMD algorithm to a typical analog signal and compares its performance with other decomposition algorithms. The signal is similar to those in references [15,29], but its composition is more complex. It contains high-frequency weak signal with intermittent time, periodic impulse signal and combined components with similar frequency. The signal can be calculated via Equation (31).

(31) $$\left\{ \begin{array}{l} x(t)=x_{1}(t)+x_{2}(t)+x_{3}(t)+x_{4}(t)\\ x_{1}(t)=\sin(2\pi f_{1}t)\\ x_{2}(t)=0.6\times \sin(2\pi f_{2}t)\\ x_{3}(t)=0.4\times \sin(2\pi f_{3}t)\begin{array}{c} \begin{array}{cc} & t\in [0.1,0.2]\cup [0.8,0.9] \end{array} \end{array}\\ x_{4}(t)=\sum_{i}A_{i}h(t-iT-\nu_{i}) \end{array}\right.$$

**Figure 3:**
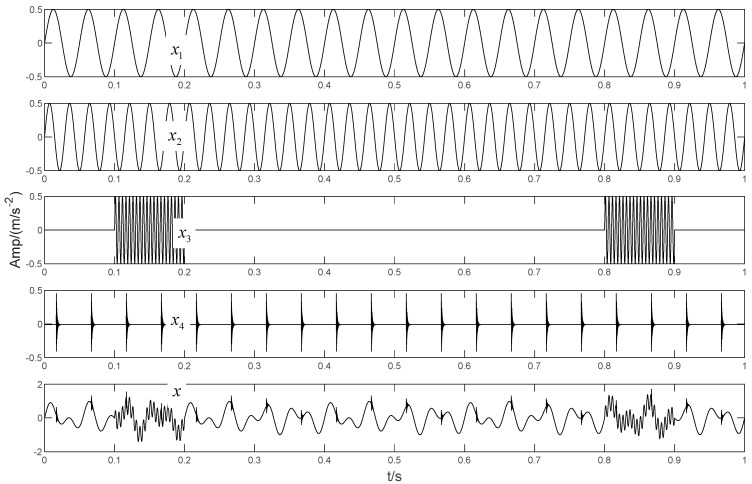
The simulated signal.

The simulated signal x(t) is composed of three sinusoidal signals $x_{1}(t)$, $x_{2}(t)$ and $x_{3}(t)$ with different center frequencies, and a high frequency intermittent signal $x_{4}(t)$, as shown in Figure 3. Here, the sampling frequencies are $f_{1}=20\;\mathrm{Hz}$, $f_{2}=35\;\mathrm{Hz}$ and $f_{3}=200\;\mathrm{Hz}$, respectively. $x_{4}(t)$ is a sinusoidal signal with periodic pulse attenuation and a frequency of 8 Hz, and is formed as:

(32) $$\left\{ \begin{array}{l} x_{4}(t)=\sum_{i}A_{i}h(t-iT-\nu_{i})\\ h(t)=e^{-Ct}\sin(2\pi f_{n}t)\\ A_{i}=1+A_{0}\sin(2\pi f_{r}t) \end{array}\right.$$

where $A_{0}$ is the initial amplitude of the impulse signal and $A_{i}$ is the amplitude of shock signal after the i-th attenuation. T is the cyclic period, $\nu_{i}$ is the random tiny slippage during each T, usually considered as 0.01T–0.02T. C denotes the damping coefficient. $f_{r}$ is the rotation frequency of the simulated transmission shaft and $f_{n}$ is the resonance frequency. Here, the simulated signal parameters are set as follow: C = 750, $f_{r}$ = 8 Hz, $f_{n}$ = 3000 Hz, $A_{0}$ = 0.5.

**Figure 4:**
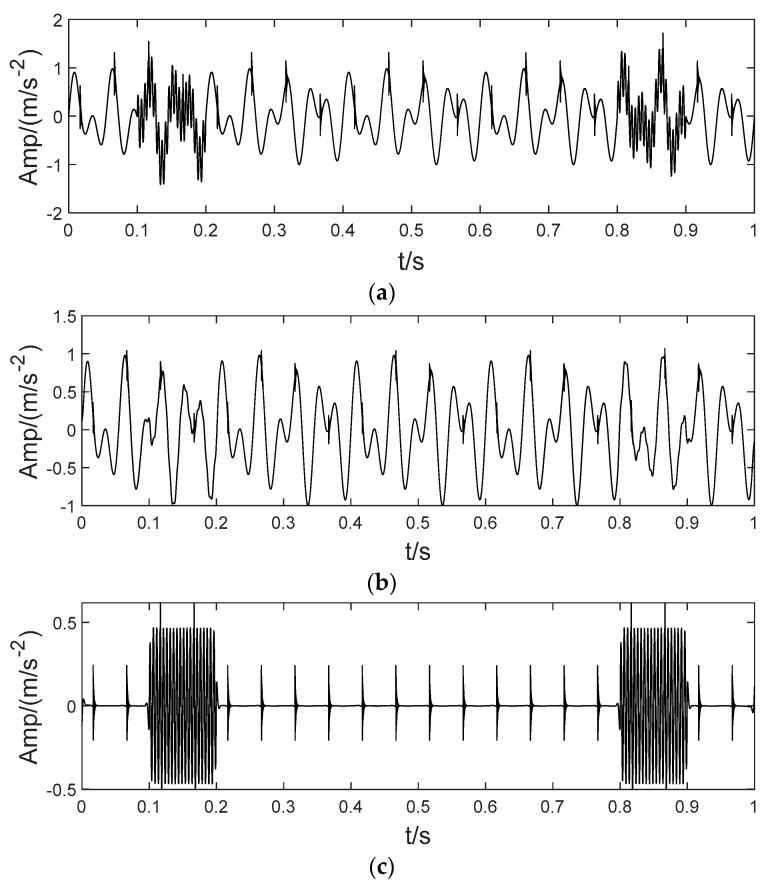
The first decomposition of empirical VMD based on binary tree. (**a**) Original simulation signal. (**b**) denotes the IMF1 component obtained by the first decomposition and (**c**) denotes IMF2.

**Figure 5:**
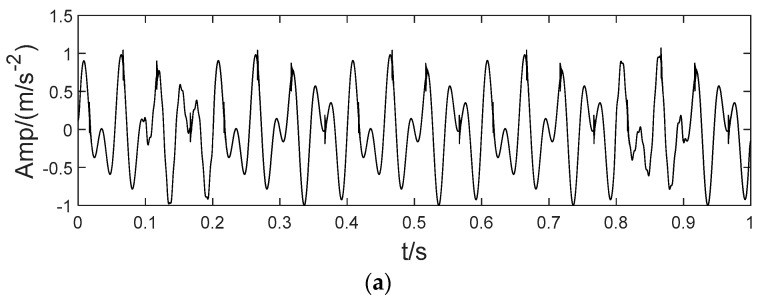

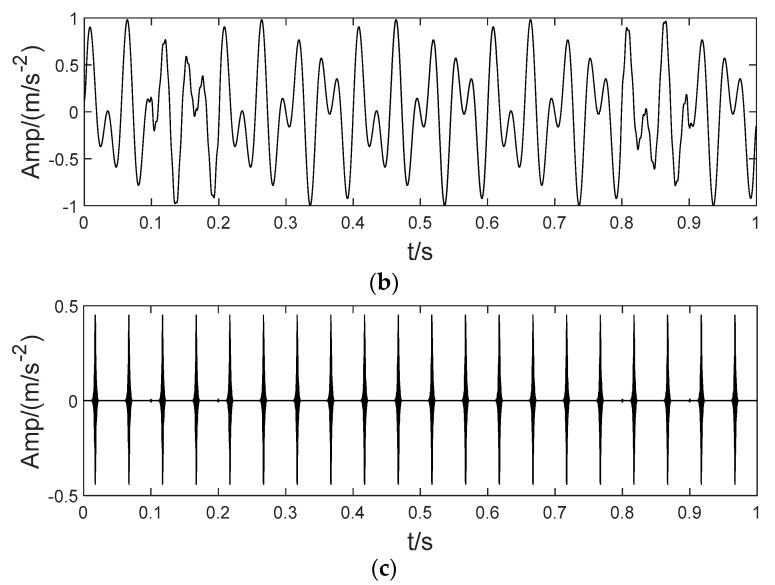
The second decomposition of IMF1. (**a**) The IMF1, (**b**) The IMF11 obtained by decomposition and (**c**) IMF12.

**Figure 6:**
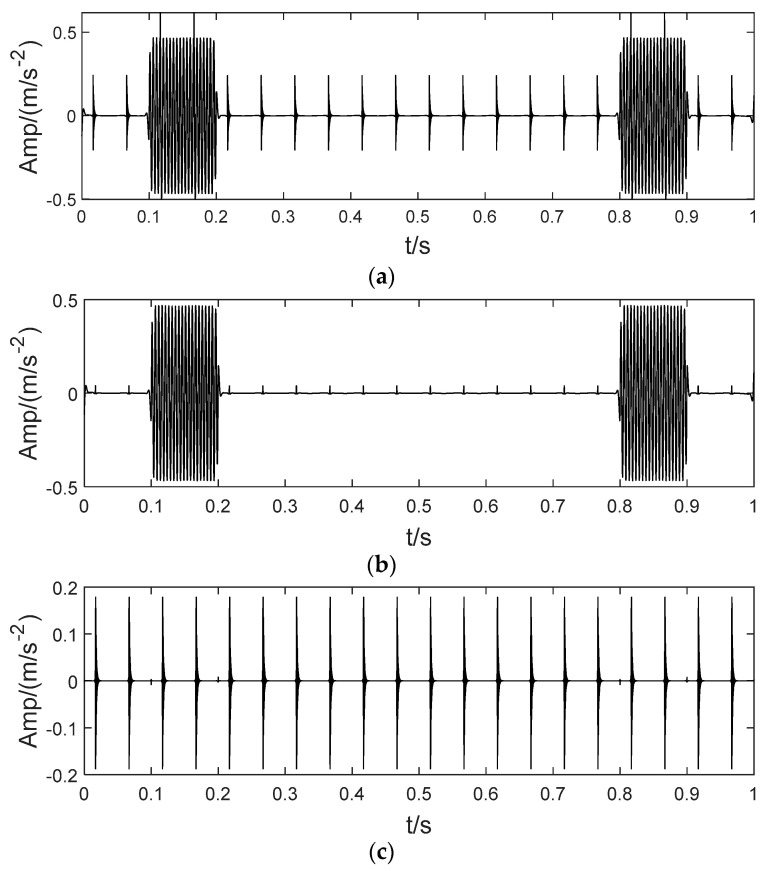
The second decomposition of IMF2. (**a**) IMF2, (**b**) The IMF21 obtained by decomposition and (**c**) IMF22.

**Figure 7:**
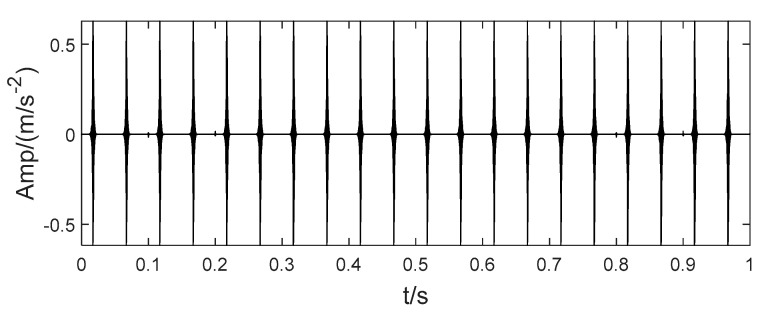
The first obtained IMF component.

**Figure 8:**
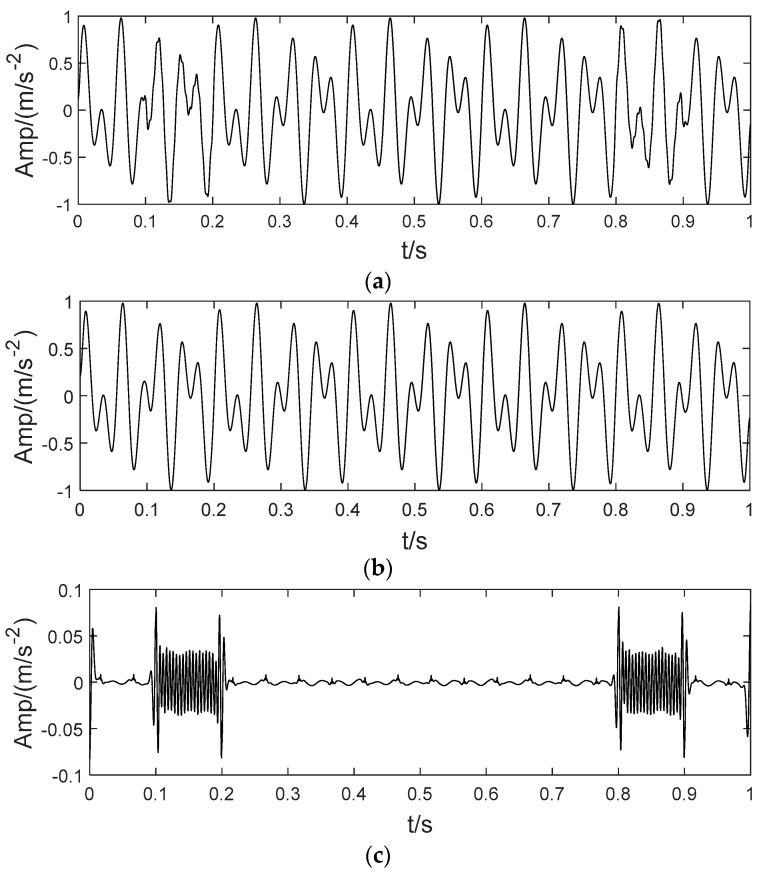
The Third decomposition of IMF11. (**a**) IMF11, (**b**) The IMF111 obtained by decomposition and (**c**) IMF112.

**Figure 9:**
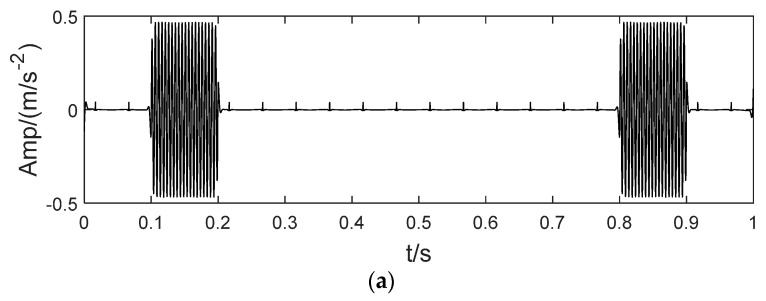

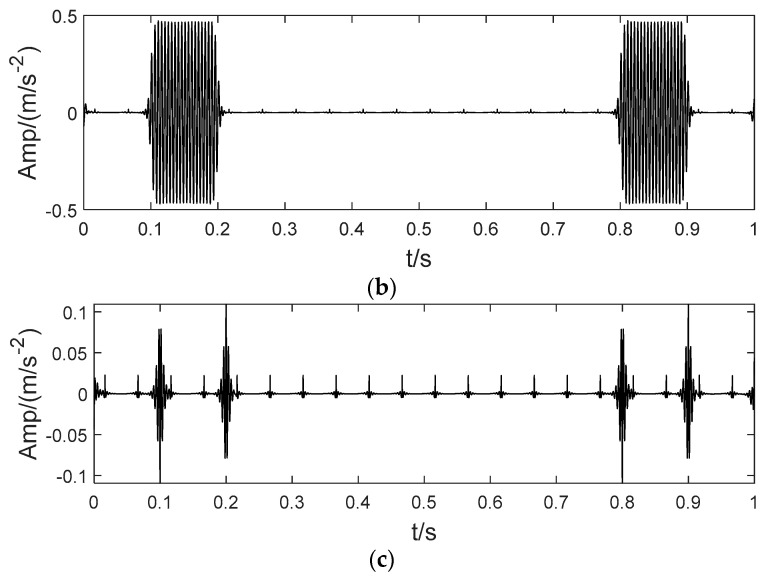
The third decomposition result. (**a**) IMF21, (**b**) IMF211 and (**c**) IMF212.

**Figure 10:**
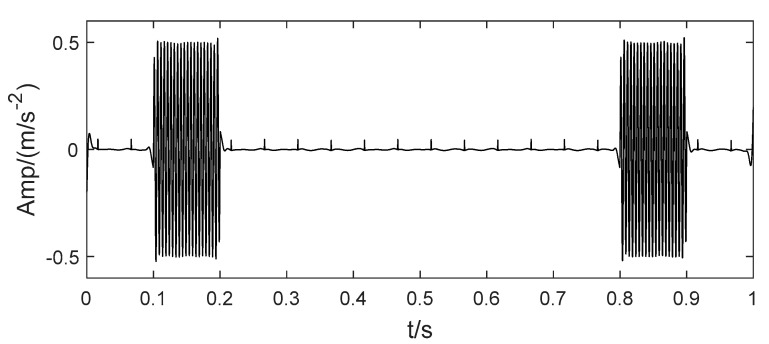
The second IMF component obtained by decomposition.

**Figure 11:**
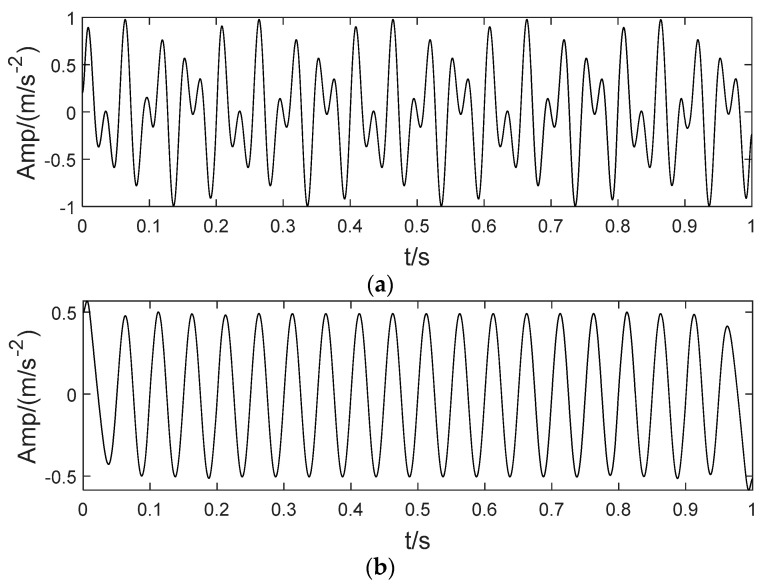

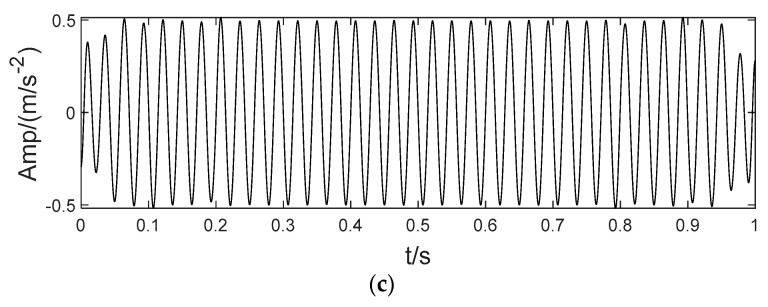
The fourth decomposition result. (**a**) IMF111, (**b**) IMF1111 and (**c**) IMF1112.

**Figure 12:**
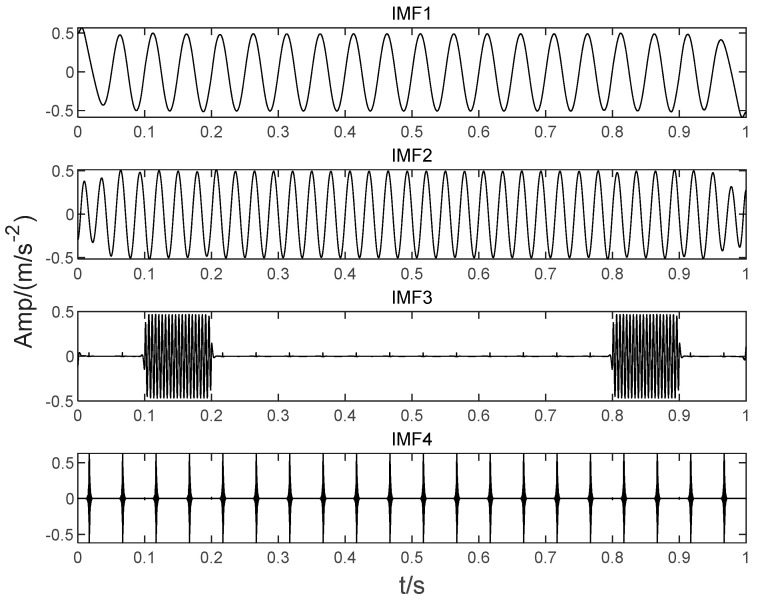
Decomposition of the mixed signal by proposed BT-EVMD method.

In order to illustrate the process of the algorithm, the binary tree empirical VMD algorithm is used to decompose the simulation signal presented in Figure 3, and the experimental results are shown in Figure 4, Figure 5, Figure 6, Figure 7, Figure 8, Figure 9, Figure 10 and Figure 11. The simulated signal is firstly decomposed into two modes: IMF1 and IMF2. According to experimental experience, the threshold of decomposition termination is set as δ = 0.1 and ρ = 0.1, respectively. Then, the LSMI and RSE between IMF1 and IMF2 are computed, and the results are shown in Table 1. The parameters listed in Table 1 are LSMI [IMF1, IMF2] = 0.0329 and RSE = 0.0328, respectively. The result of the comparison indicates that the first VMD decomposition of the simulated signal does not meet the stop condition. Therefore, components IMF1 and IMF2 need to be further decomposed, that is, the second-layer VMD decomposition. The decomposition results are shown in Figure 5 and Figure 6, in which the waveforms of components IMF12 and IMF22 are actually very similar. In order to quantify the similarity between the two components, the LSMI is computed as LSMI [IMF12, IMF22] = 0.8414, which satisfies the stop condition. At this time, the components IMF12 and IMF22 do not need to be further decomposed, and the first component IMF1’ is obtained by adding the modes IMF12 and IMF22, as shown in Figure 7. The remaining components will then be decomposed by VMD. The third level decomposition results are shown in Figure 8 and Figure 9. The component IMF11 is decomposed into sub-components IMF111 and IMF112, and the sub-modes IMF211 and IMF212 are obtained by decomposing the IMF21. The corresponding computation parameters of RSE and LSMI between sub-components can be found in Table 1. The Components IMF112, IMF211, and IMF212 are selected as their values of LSMI satisfy the stop condition. Thus, IMF112, IMF211 and IMF212 are added to construct a new mode IMF2’, and the resultant signal is shown in Figure 10. Then, the remnant component IMF111 is decomposed, and the decomposition result is shown in Figure 11. It can be seen from Figure 11 that the decomposed components IMF1111 and IMF1112 are two single wave modes, so the decomposition is stopped and the two components are regarded as the third component IMF3’ and the fourth component IMF4’ respectively. Finally, four modal components are obtained, as shown in Figure 12. It can be observed from the experimental results that the number of modal components obtained by the proposed method is the same as that of the original simulation signal, and the waveform features are highly similar. To further explain the quantitative relationship between each IMF component obtained and its corresponding original simulated signal, the LSMI is computed between them. The specification of the LSMI is listed in Table 1. From the measured data, except that the LSMI between the component IMF4′ and the simulated signal $x_{4}$ is slightly less than 1, other IMF components are almost the same as their corresponding simulated signals. Experimental results indicate that the proposed empirical VMD algorithm is adaptive and effective.

**Figure 13:**
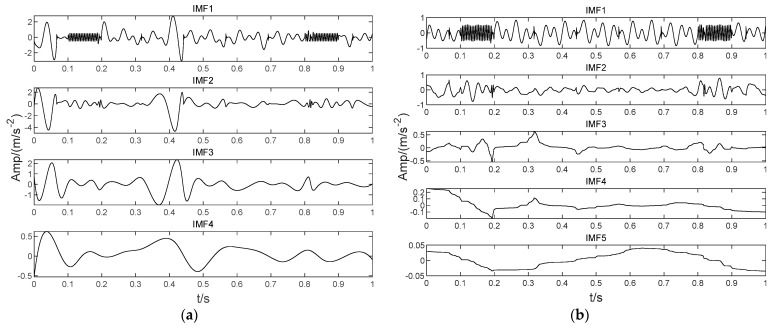

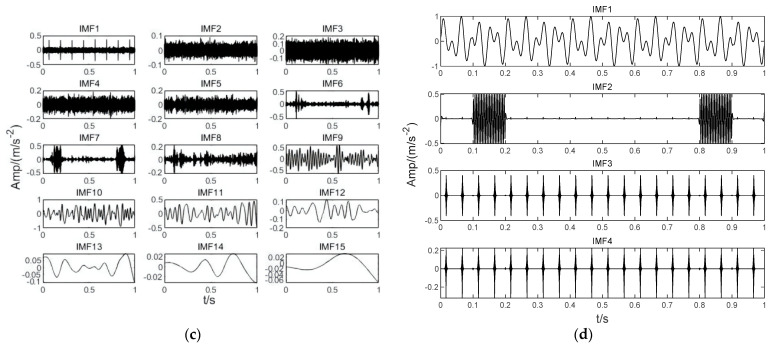
Performance evaluation of other state-of-art methods. (**a**) The components obtained by LMD. (**b**) The components obtained by ITD. (**c**) The components obtained by CEEMDAN. (**d**) The components obtained by SVMD.

In order to verify the superiority of the proposed algorithm, this paper continues to utilize common signal processing methods such as LDM, ITD, CEEMDAN and SVMD to process the simulation signal. The decomposition mode results are shown in Figure 13. Figure 13a denotes the result of the LMD decomposition. The signal is decomposed into four independent components, the number of which corresponds to that of the simulation signal without obvious aliasing, but the components corresponding to the simulation signal have no similarity at all. Figure 13b is the result of the ITD decomposition. The number of components decomposed by this method is inconsistent with that of the simulation signal, and there is no similarity with the original components. The number of components decomposed by the CEEMDAN method far exceeds the number of original signals, and there is a certain amount of mode mixing. Some waveform trends have certain similarities with the components of the original signal (such as IMF1−IMF3), but there are many irrelevant terms due to the over decomposition phenomenon, and the results are shown in Figure 13c. Figure 13d is the decomposition result of SVMD. Comparing the waveform of the decomposed components with the original components, it can be observed that the decomposed IMF1 is a mixed signal of the $x_{1}(t)$ and $x_{2}(t)$ in the simulation signal, that is, the first IMF component is not a single component. In addition, the waveforms of the IMF3 and the IMF4 are similar, indicating that over decomposition occurs. Therefore, comparing the decomposition results of Figure 12 and Figure 13, it can be observed that the components obtained based on the method proposed in this paper are not only consistent with the simulated signal in quantity, but also the characterization of the components is almost the same as that of the simulated signal. Compared with the above methods, the experimental results indicate that the performance of the proposed method has obvious advantages.

### 4.2. Analysis of Test Data 

**Figure 14:**
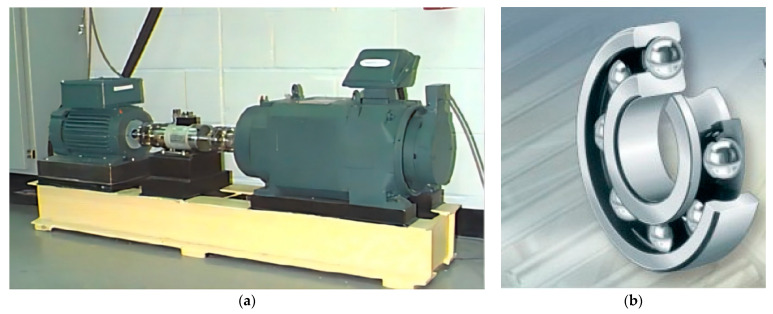
Apparatus & procedures: (**a**) Experimental platform. (**b**) Skf6205 deep groove ball bearing.

In order to further verify the effectiveness of the proposed method in actual signal analysis, in this paper, the bearing fault signal of Western Reserve University was applied as the verification data [42]. As shown in Figure 14, the bearing fault test rig mainly consists of an induction motor (2 HP), a torque transducer, a dynamometer, and several units. Three accelerometers are mounted on the housing at 3, 6 and 12 o’clock positions of the motor drive ports. The vibration signals of rolling bearings were collected by a 16-channel data recorder. The platform used electrical discharge machining to arrange a single point of failure on the bearing (SKF6205).

In the experiment, the fault data of the inner ring and outer ring of the drive end bearing were collected for analysis. According to Ref. [43], the specification parameters are listed in Table 2.

**Figure 15:**
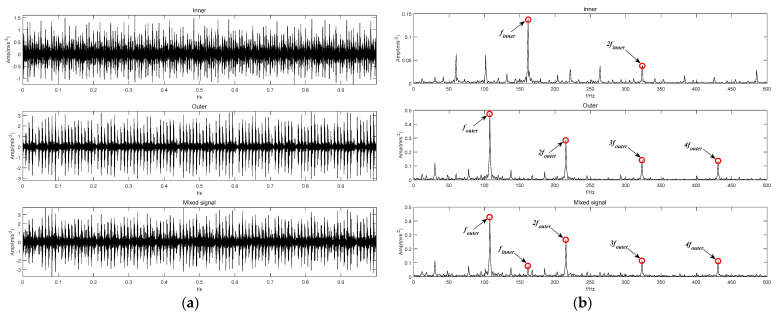
Original fault vibration signal: (**a**) Time-domain fault waveform. (**b**) Envelope spectrum.

**Figure 16:**
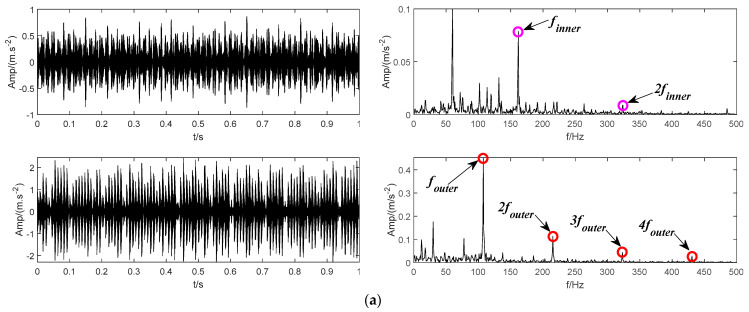

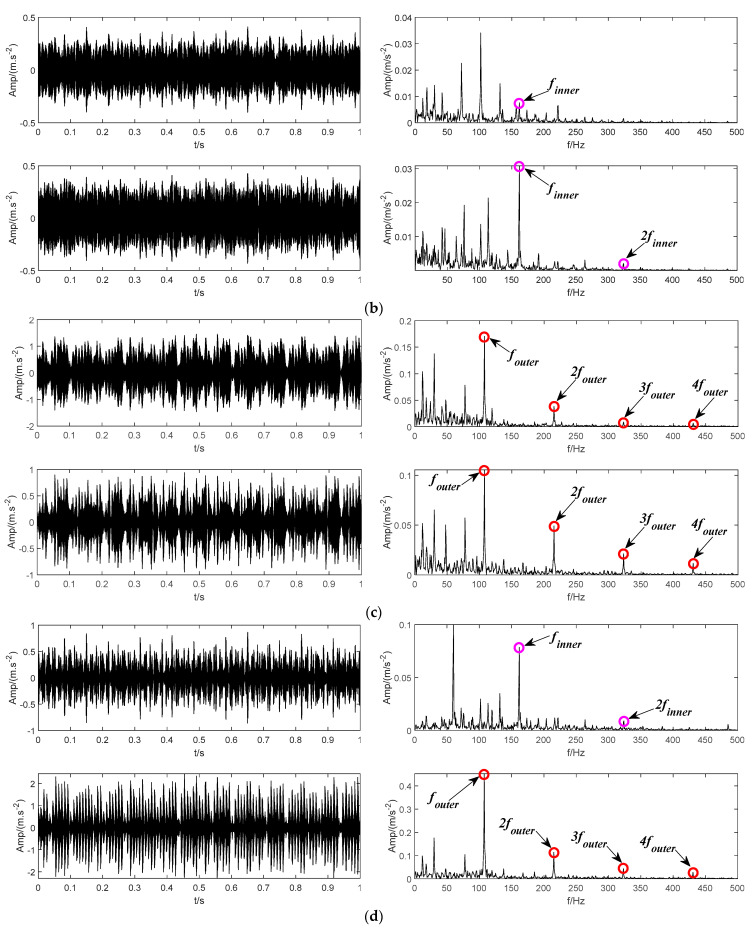
The modes and the corresponding envelope spectrum obtained by the proposed BT-EVMD method. (**a**) The first decomposition. (**b**) The second decomposition. (**c**) The third decomposition. (**d**) The final result.

**Figure 17:**
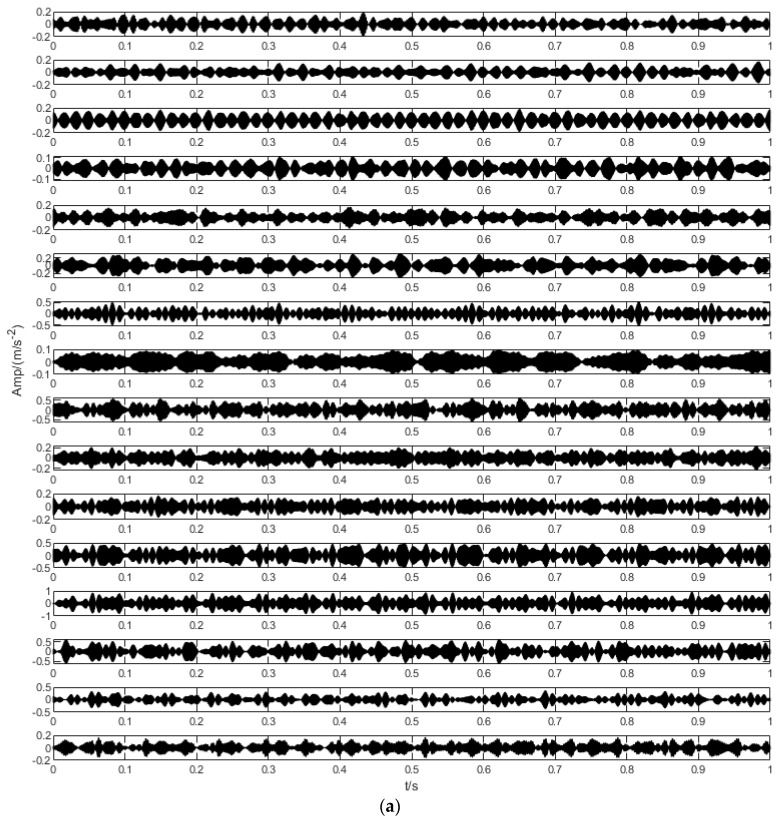

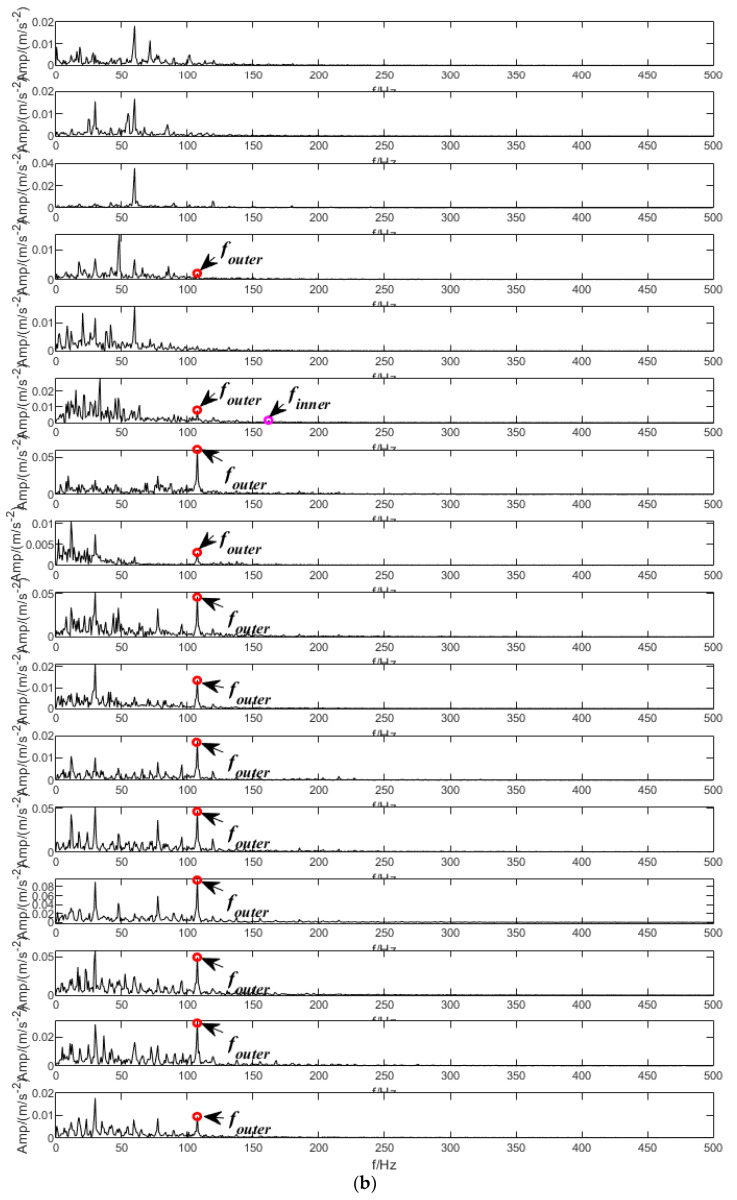
The IMF components and envelope spectrums using SVMD. (**a**) Decomposed modes. (**b**) Envelope spectrums.

**Figure 18:**
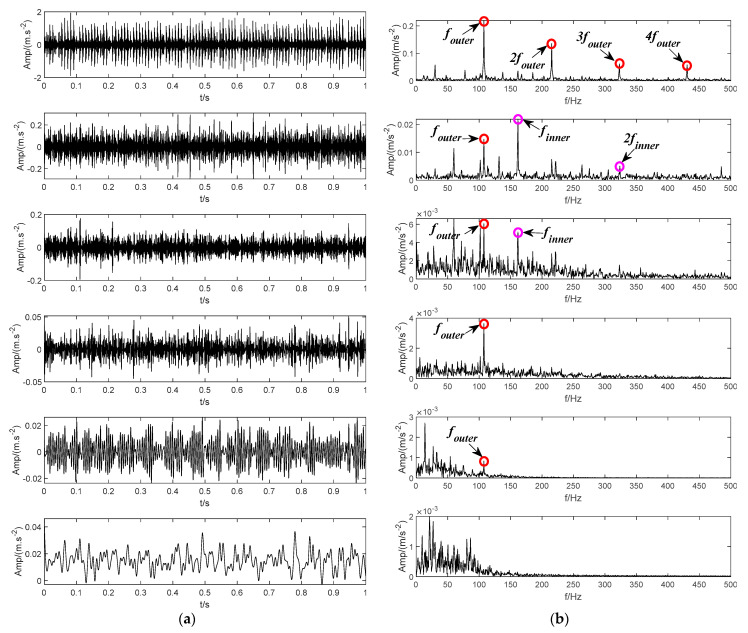
Decomposition result of CEEMDAN method. (**a**) The IMF components. (**b**) Envelope spectrums.

**Figure 19:**
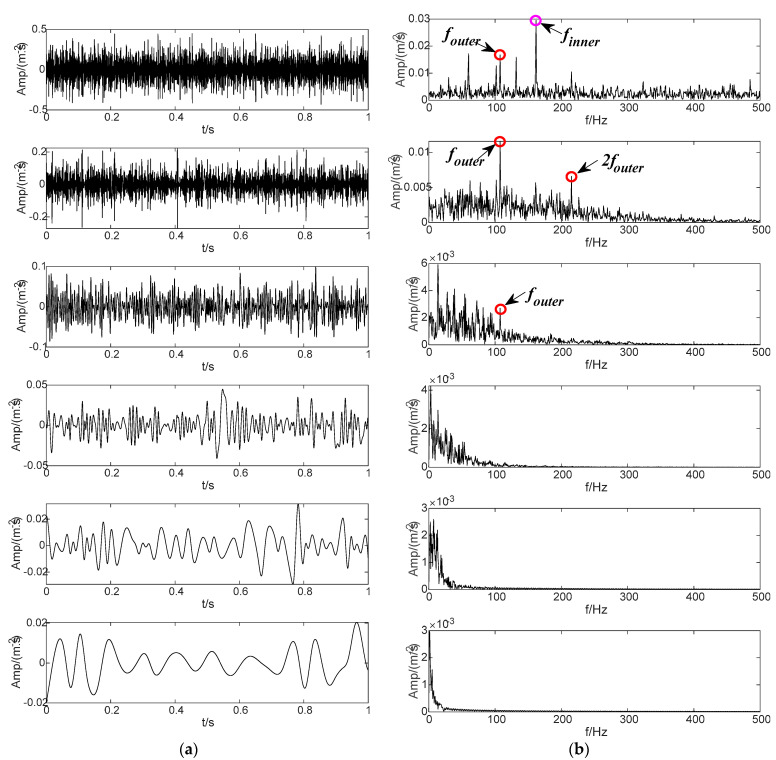
Decomposition result of LMD method. (**a**) The PF components obtained by LMD and (**b**) their envelope spectrums.

**Figure 20:**
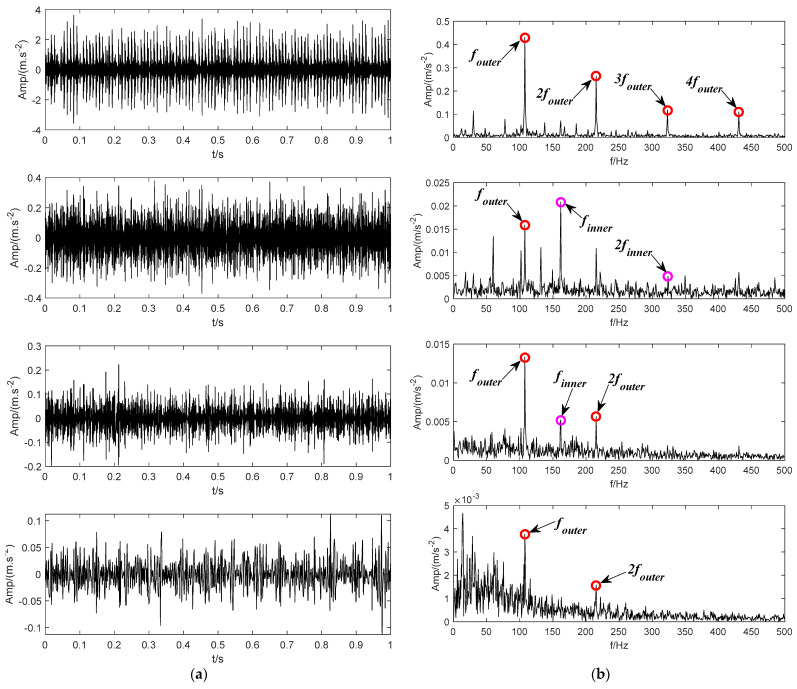
Performance evaluation of ITD method. (**a**) The PRC components obtained by ITD and (**b**) their envelope spectrums.

According to the method applied in [24], the inner and outer ring bearing fault signals are superimposed to obtain a mixed fault signal, as shown in Figure 15. Figure 15a shows the time domain waveforms of inner and outer ring bearing fault signals and the mixed signal. Figure 15b corresponds to their envelope spectrum analysis, respectively. The inner ring fault characteristic frequency $f_{inne}{}_{r}$, outer ring fault characteristic frequency $f_{outer}$ and its frequency doubling items can be observed in the spectrum of the mixed signal. Then, the mixed signal is analyzed by using SVMD, CEEMDAN, LDM, ITD and the proposed BT-EVMD methods. The decomposition results are shown in Figure 16, Figure 17, Figure 18, Figure 19 and Figure 20, respectively.

The decomposition result of the proposed BT-EVMD is shown in Figure 16. The relevant decomposition parameters are shown in Table 3. The decomposition results of the first layer are shown in Figure 16a, in which the left part is the two IMF components obtained, and the right part is the envelope spectrums corresponding to the IMF component. From the envelope spectrum corresponding to IMF1 and IMF2 components, it can be observed that the envelope spectrum of IMF1 component includes the fault characteristic frequency $f_{inner}$ and its doubling frequency $2f_{inner}$ of the bearing inner ring. Correspondingly, the envelope spectrum of IMF2 contains the fault characteristic frequency of the bearing outer ring (fundamental frequency , harmonic components 2$f_{outer}$, 3 and 4$f_{outer}$). The decomposition results of the first layer show that the fault signals representing the inner and outer rings of the bearing can be significantly separated. The mutual information results in Table 3 also verify the effectiveness of this decomposition. Although the mutual information between the two IMFs is very small, it cannot be proved that each mode is a mono-component signal. According to the decomposition process of BT-EVMD, the termination condition needs to be further verified. Thus, the two IMFs are further decomposed, and the results are shown in Figure 16b,c, respectively.

From the time domain components decomposed by IMF1 and IMF2 and their corresponding envelope spectra, it can be observed that the spectrum of IMF11 and IMF12 contain the fault characteristic frequency of the bearing inner ring, and the envelope spectra of IMF21 and IMF22 primarily contain the fault characteristic frequency of the bearing outer ring. The results in Table 2 show that the mutual information of the signals decomposed by IMF1 and IMF2 is relatively large. The situation indicates that the components are duplicated in the process of signal decomposition and there is no need to decompose. Afterwards, the components of the second layer decomposition are superimposed, that is, the original IMF1 and IMF2 components are restored. The final decomposition results are shown in Figure 16d.

To evaluate the decomposition effect, some commonly used signal decomposition methods are used for signal processing and comparative analysis. Figure 17 shows the decomposition result based on the SVMD method, which finally obtains 16 IMF components. By observing the envelope spectrum corresponding to IMF components, it can be seen that many IMF components have common frequency items, indicating that they have copied themselves. The envelope spectrum of IMF6 component includes the fault characteristic frequencies of the inner and outer rings of the bearing at the same time. It shows that this method cannot effectively decompose the fault signals of the inner and outer rings in the signal. Figure 18 shows the decomposition results based on the CEEMDAN method, and six independent IMF components are obtained by this method. From the envelope spectrum analysis corresponding to each IMF component, it can be observed that some IMF components replicate each other. In particular, the envelope spectra of IMF2 and IMF3 components include the fault characteristic frequencies of the inner and outer rings of the bearing at the same time. In other words, there is a certain mixing feature. It shows that this method lacks the ability to effectively distinguish the internal and external fault characteristics in the signal to some extent. Based on the decomposition of the LMD method, six PF components are obtained, and the results are shown in Figure 19. The observation shows that some PF components are duplicated, and the envelope spectrum of the PF1 component includes the fault characteristic frequencies of the inner and outer rings of the bearing. The results show that the LMD method still underperforms in the effective differentiation of fault items in the signal. The decomposition result of the ITD method is shown in Figure 20, which finally obtains four PRC components. In the component envelope spectrums shown in Figure 20b, PRC1 includes the fault characteristic frequency of the inner ring bearing, and the frequency spectrum of PRC4 contains the fault characteristic frequency of the bearing outer ring. However, the PRC2 and the PRC3 contain both inner and outer ring fault characteristic frequencies of the bearing. The results show that this method does not effectively distinguish the fault items in the signal. The comparative experimental results show that the BT-EVMD is more effective and robust than the above four methods.

## 5. Discussion and Conclusions

Novelties and Contributions: In this paper, an empirical VMD algorithm based on binary tree is proposed. By introducing the binary tree model and merging it into the VMD algorithm, the problem of selecting the total modal K of VMD can be effectively avoided. The binary tree model is a type of ergodic model that needs to select suitable stop conditions. In this paper, the least square mutual information is introduced as one of the stopping conditions of the VMD algorithm, which can effectively measure the nonlinear coupling degree of information between two IMF components and guarantee the orthogonality between IMF components. In addition, the performance of VMD is also affected by the value of the parameter [α,τ]. By analyzing the influence of parameter [α,τ] on the performance of the VMD algorithm, the RCMDE of the signal is empirically introduced to dynamically adjust the value of parameter α, and the SNR of the signal is taken as the value of parameter τ. These improvements not only effectively avoid the experiential, blind and accidental problems of artificially setting parameters, they also avoid the rationality of designing fitness functions when searching VMD key parameters with an intelligent optimization algorithm, and the problem of excessive computational complexity. Lastly, simulation and measured signals are compared and analyzed with LMD, ITD, CEEMDAN and excellent SVMD methods. Experimental results indicate that the proposed method has better decomposition performance and robustness, and has full adaptability.

Further Work: The binary tree model requires a certain iterative process and certain judgment conditions, so it has increased certain computational complexity compared to traditional VMD. In addition, the value of the VMD parameter [α,τ] is set through the computation of the signal time domain method, which is very empirical and lacks rigorous mathematical derivation and proof. Therefore, further research is required. In addition, there is a drawback when using RCMDE to dynamically adjust parameter α. That is, when the number of components in the signal is tiny, and their center frequency is too near, the value of RCMDE will lead to the value of α being too small, and the components whose center frequency is too close will be divided into the same frequency band, hence generating mode aliasing. Although this defect can be resolved through the iterative process of the binary tree, it will expend a large amount of computation. As a result, the value of parameter α and its solution need to be further researched.

## Figures and Tables

**Table 1:** The LSMI and RSE obtained by BT-EVMD.

Decomposition Hierarchy	Modes	LSMI Index	Reconstruction Error
The first layer	[IMF1, IMF2]	LSMI [IMF1, IMF2] = 0.0329	RSE = 0.0328
The second layer	[IMF11, IMF12] [IMF21, IMF22]	LSMI [IMF11, IMF12] = 0.0002 LSMI [IMF21, IMF22] = 0.0016 LSMI [IMF11, IMF21] = 0.0028 LSMI [IMF11, IMF22] = 0.0004 LSMI [IMF12, IMF21] = 0.0012 LSMI [IMF12, IMF22] = 0.8414	RSE = 0.0414
The third layer	[IMF111, IMF112] [IMF211, IMF212]	LSMI [IMF111, IMF112] = 0.1441 LSMI [IMF211, IMF212] = 0.0849 LSMI [IMF111, IMF211] = 0.0052 LSMI [IMF111, IMF212] = 0.0228 LSMI [IMF112, IMF211] = 0.7181 LSMI [IMF112, IMF212] = 0.1325	RSE = 0.0327
The fourth layer	[IMF1111, IMF1112]	LSMI [IMF1111, IMF1112] = 0.0185	RSE = 0.0112

**Table 2:** The test platform parameters.

Bearing Fault Type	Parameters				
	Speed (r/min)	Characteristic Frequency (Hz)	Load (HP)	Data Length (L)	Sampling Frequency (kHz)
Inner ring fault	1797	162.1852	0	12,000	12
Outer ring fault	1796	107.3647	0	12,000	12

**Table 3:** The measurements obtained by BT-EVMD.

Decomposition Level	Parameters	Components	LSMI	Reconstruction Error
The first layer	K = 2, α1 = 16,363, τ = 0.019	[IMF1, IMF2]	LSMI [IMF1, IMF2] = 0.0058	RSE = 0.0008
The second layer	K1 = 2, α1 = 21,386, τ1 = 0.011	[IMF11, IMF12]	LSMI [IMF11, IMF12] = 0.1849	RSE1 = 0.0004
	K2 = 2, α2 = 5258, τ2 = 0.010	[IMF21, IMF22]	LSMI [IMF21, IMF22] = 0.1916	RSE2 = 0.0002

## Data Availability

Not applicable.

## References

[1] He M., He D. (2017). Deep Learning Based Approach for Bearing Fault Diagnosis. IEEE Trans. Ind. Appl..

[2] Wang H., Peng C. (2010). Fuzzy Diagnosis Method for Rotating Machinery in Variable Rotating Speed. IEEE Sens. J..

[3] Chen J., Pan J., Li Z., Zi Y., Chen X. (2016). Generator bearing fault diagnosis for wind turbine via empirical wavelet transform using measured vibration signals. Renew. Energy.

[4] Yan R., Gao R.X., Chen X. (2014). Wavelets for fault diagnosis of rotary machines: A review with applications. Signal Process..

[5] Liu X., Jia Y.X., He Z.W., Zhou J. (2017). Application of EMD-WVD and particle filter for gearbox fault feature extraction and remaining useful life prediction. J. Vibroeng..

[6] Faysal A., Ngui W.K., Lim M.H., Leong M.S. (2021). Noise Eliminated Ensemble Empirical Mode Decomposition Scalogram Analysis for Rotating Machinery Fault Diagnosis. Sensors.

[7] Wang Y.H., Rao Y., Xu D. (2020). Multichannel maximum-entropy method for the Wigner-Ville distribution. Geophysics.

[8] Zhang X.H., Zhao J.M., Bajrić R., Wang L.L. (2017). Application of the DC Offset Cancellation Method and S Transform to Gearbox Fault Diagnosis. Appl. Sci..

[9] Peppas K.P., Mathiopoulos P.T., Yang J., Zhang C., Sasase I. (2018). High-Order Statistics for the Channel Capacity of EGC Receivers Over Generalized Fading Channels. IEEE Commun. Lett..

[10] Guo T., Deng Z.M. (2017). An improved EMD method based on the multi-objective optimization and its application to fault feature extraction of rolling bearing. Appl. Acoust..

[11] Li C.W., Zhan L.W., Shen L.Q. (2015). Friction Signal Denoising Using Complete Ensemble EMD with Adaptive Noise and Mutual Information. Entropy.

[12] Colominas M.A., Schlotthauer G., Torres M.E. (2014). Improved complete ensemble EMD: A suitable tool for biomedical signal processing. Biomed. Signal Process. Control.

[13] Osman S., Wang W. (2016). A Morphological Hilbert-Huang Transform Technique for Bearing Fault Detection. IEEE Trans. Instrum. Meas..

[14] Gao S.P., Xu Z.X., Song G.B., Shao M.X., Jiang Y.Y. (2021). Fault Location of Hybrid Three-terminal HVDC Transmission Line Based on Improved LMD. Electr. Pow. Syst. Res..

[15] Song E.Z., Gao F., Yao C., Ke Y. (2021). Research on Rolling Bearing Fault Diagnosis Method Based on Improved LMD and CMWPE. J. Fail. Anal. Prev..

[16] Priyadarshini L., Dash P.K. (2021). Detection of Islanding and Non-islanding Fault Disturbances in Microgrid Using LMD and Deep Stacked RVFLN Based Auto-encode. Electr. Eng..

[17] Yu M., Pan X. (2020). A Novel ITD-GSP-based Characteristic Extraction Method for Compound Faults of Rolling Bearing. Measurement.

[18] Voznesensky A., Kaplun D. (2019). Adaptive Signal Processing Algorithms Based on EMD and ITD. IEEE Access.

[19] Dragomiretskiy K., Zosso D. (2014). Variational Mode Decomposition. IEEE Trans. Signal Process..

[20] Li K., Su L., Wu J.J., Wang H.Q., Chen P. (2017). A Rolling Bearing Fault Diagnosis Method Based on Variational Mode Decomposition and an Improved Kernel Extreme Learning Machine. Appl. Sci..

[21] Zhang M., Jiang Z.N., Feng K. (2017). Research on variational mode decomposition in rolling bearings fault diagnosis of the multistage centrifugal pump. Mech. Syst. Signal Process..

[22] Mei L., Li S.Y., Zhang C., Han M.X. (2021). Adaptive Signal Enhancement Based on Improved VMD-SVD for Leak Location in Water-Supply Pipeline. IEEE Sens. J..

[23] Liu C.F., Zhu L.D., Ni C.B. (2018). Chatter detection in milling process based on VMD and energy entropy. Mech. Syst. Signal Process..

[24] He X.Z., Zhou X.Q., Yu W.N., Hou Y.X., Mechefske C.K. (2021). Adaptive Variational Mode Decomposition and Its Application to Multi-fault Detection Using Mechanical Vibration Signals. ISA Trans..

[25] Xiao H.S., Wei J.C., Liu H.S., Li Q.Q., Shi Y.L. (2017). Identification method for power system low-frequency oscillations based on improved VMD and Teager-Kaiser energy operator. IET Gener. Transm. Distrib..

[26] Kaur C., Bisht A., Singh P., Joshi G. (2021). EEG Signal denoising using hybrid approach of Variational Mode Decomposition and wavelets for depression. Biomed. Signal Process. Control.

[27] Long J.C., Wang X.P., Dai D.D., Tian M., Zhu G.W., Zhang J. (2017). Denoising of UHF PD signals based on optimised VMD and wavelet transform. IET Sci. Meas. Technol..

[28] Li Y., Xu F.Y. (2022). Acoustic emission sources localization of laser cladding metallic panels using improved fruit fly optimization algorithm-based independent variational mode decomposition. Mech. Syst. Signal Process..

[29] Wang X.B., Yang Z.X., Yan X.A. (2017). Novel particle swarm optimization-based variational mode decomposition method for the fault diagnosis of complex rotating machinery. IEEE/ASME Trans. Mechatron..

[30] Xu B., Zhou F.X., Li H.P., Yan B.K., Liu Y. (2019). Early fault feature extraction of bearings based on Teager energy operator and optimal VMD. ISA Trans..

[31] Nazari M., Sakhaei S.M. (2018). Variational Mode Extraction: A New Efficient Method to Derive Respiratory Signals from ECG. IEEE J. Biomed. Health Inform..

[32] Nazari M., Sakhaei S.M. (2020). Successive Variational Mode Decomposition. Signal Process..

[33] Liu S.S., Yu K.P. (2022). Successive Multivariate Variational Mode Decomposition Based on Instantaneous Linear Mixing Model. Signal Process..

[34] Chen Q.M., Chen J.M., Lang X., Xie L., Rehman N.U., Su H.Y. (2021). Self-tuning Variational Mode Decomposition. J. Frankl. Inst..

[35] Pahlavani P., Bigdeli B. (2017). A mutual information Dempster-Shafer based decision ensemble system for land cover classification of hyperspectral data. Front. Earth Sci..

[36] Valdez M.A., Jaschke D., Vargas D.L., Carr L.D. (2017). Quantifying complexity in quantum phase transitions via mutual information complex networks. Phys. Rev. Lett..

[37] Ball K.R., Grant C., Mundy W.R., Shafer T.J. (2017). A multivariate extension of mutual information for growing neural networks. Neural Netw..

[38] Tani N., Ohta M., Higuchi Y., Akatsu J., Kumashiro M. (2020). Lifestyle and subjective musculoskeletal symptoms in young male Japanese workers: A 16-year retrospective cohort study. Prev. Med. Rep..

[39] Sukriti, Chakraborty M., Mitra D. (2021). Automated detection of epileptic seizures using multiscale and refined composite multiscale dispersion entropy. Chaos Solitons Fractals.

[40] Rostaghi M., Azami H. (2016). Dispersion Entropy: A Measure for Time-Series Analysis. IEEE Signal Process. Lett..

[41] Azami H., Rostaghi M., Abasolo D., Escudero J. (2017). Refined Composite Multiscale Dispersion Entropy and its Application to Biomedical Signals. IEEE Trans. Biomed. Eng..

[42] CWRU Bearing Data Center. http://csegroups.case.edu/bearingdatacenter/home.

[43] Smith W.A., Randall R.B. (2015). Rolling element bearing diagnostics using the Case Western Reserve University data: A benchmark study. Mech. Syst. Signal Process..

